# Ischemic stroke associated with high‐grade pedunculated device‐related thrombosis following left atrial appendage closure

**DOI:** 10.1002/joa3.13042

**Published:** 2024-04-22

**Authors:** Ryuki Chatani, Shunsuke Kubo, Hiroshi Tasaka, Takeshi Maruo, Kazushige Kadota

**Affiliations:** ^1^ Department of Cardiovascular Medicine Kurashiki Central Hospital Kurashiki Japan

**Keywords:** atrial fibrillation, device‐related thrombosis, ischemic stroke, left atrial appendage closure, oral anticoagulation

## Abstract

We have seen ischemic stroke associated with a high‐grade device‐related pedunculated thrombosis after left atrial appendage closure (LAAC) after discontinuation of oral anticoagulations (OACs). Continuation of OACs, including half‐dose direct oral anticoagulations after LAAC, may be a better option for patients at high risk of thromboembolism to prevent further thromboembolic events.

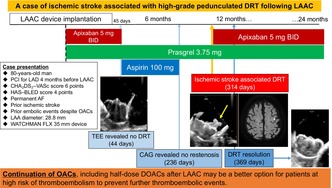

Percutaneous left atrial appendage closure (LAAC) is a viable option for patients with atrial fibrillation (AF) who cannot undergo long‐term anticoagulant therapy. Notably, LAAC has demonstrated efficacy in preventing further thromboembolic events, even in patients with continuous oral anticoagulation (OAC) failure.^1^ Recent studies have reported an incidence of device‐related thrombosis (DRT) of approximately 2%–4% within 1 year after LAAC, emphasizing the need for a classification system for DRT.^2^ In this context, we present a case involving a patient who faced two instances of OAC failure and subsequently developed high‐grade pedunculated DRT, contributing to an ischemic stroke following LAAC.^2^

An 80‐year‐old man with a history of leadless pacemaker implantation for nonvalvular permanent AF bradycardia, experienced an ischemic stroke in the absence of OACs. Despite continuous OACs, he suffered two symptomatic embolic events, had a warfarin allergy, and presented with a high bleeding risk (CHADS₂ score 4, CHA₂DS₂–VASc score 6, HAS–BLED score 4), indicating a need for LAAC. His LAA diameter was 28.8 mm. Four months before LAAC, he underwent percutaneous coronary intervention with stenting of the left anterior descending artery for angina and was prescribed prasugrel 3.75 mg daily in addition to apixaban 5 mg twice daily. LAAC was successfully performed using a 35 mm Watchman FLX (Boston Scientific), resulting in the effective sealing of the left atrial appendage. The patient was discharged without complications on postoperative day 2 (Figure 1; Video S1).

**FIGURE 1:**
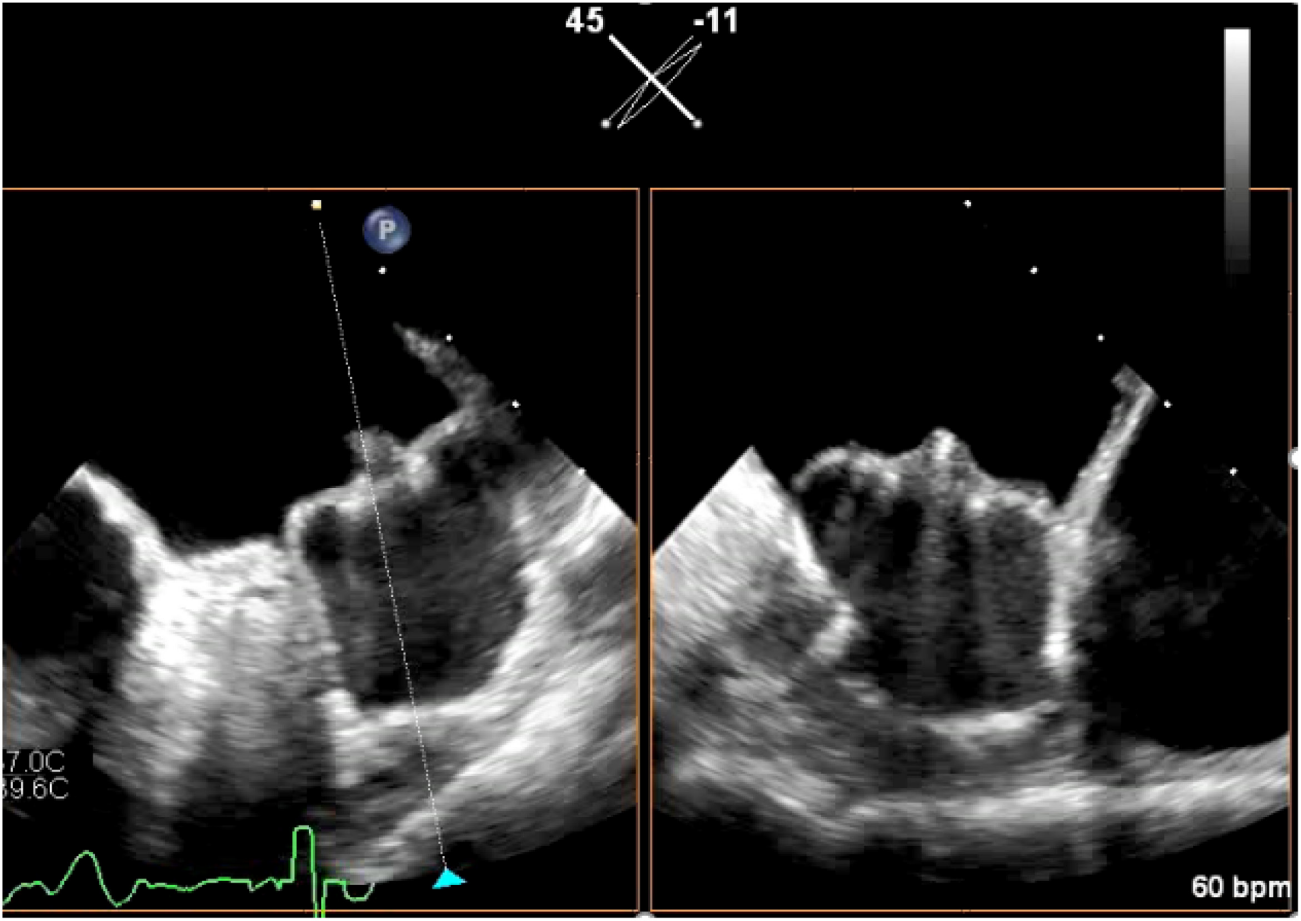
Transesophageal echocardiography image of the device after release. The device compression rate ranged 16.0%–22.9%, with no evidence of device leakage.

On postoperative day 44, transesophageal echocardiography (TEE) indicated the absence of device leakage or DRT (Videos S2). Subsequently, the patient was prescribed prasugrel 3.75 mg and aspirin 100 mg daily, with the discontinuation of apixaban according to the Japanese package insert. On postoperative day 236, follow‐up coronary angiography revealed no restenosis, leading to the decision to continue only prasugrel 3.75 mg daily, with the discontinuation of aspirin. However, on postoperative day 314, the patient was rehospitalized for left homonymous hemianopia and diagnosed with an ischemic stroke through head magnetic resonance imaging. TEE at the time revealed the presence of high‐grade pedunculated DRT (Grade 3; 17 × 20 mm) (Figure 2; Videos S3 and S4). Apixaban 5 mg twice daily was readministered, and the patient was subsequently discharged.

**FIGURE 2:**
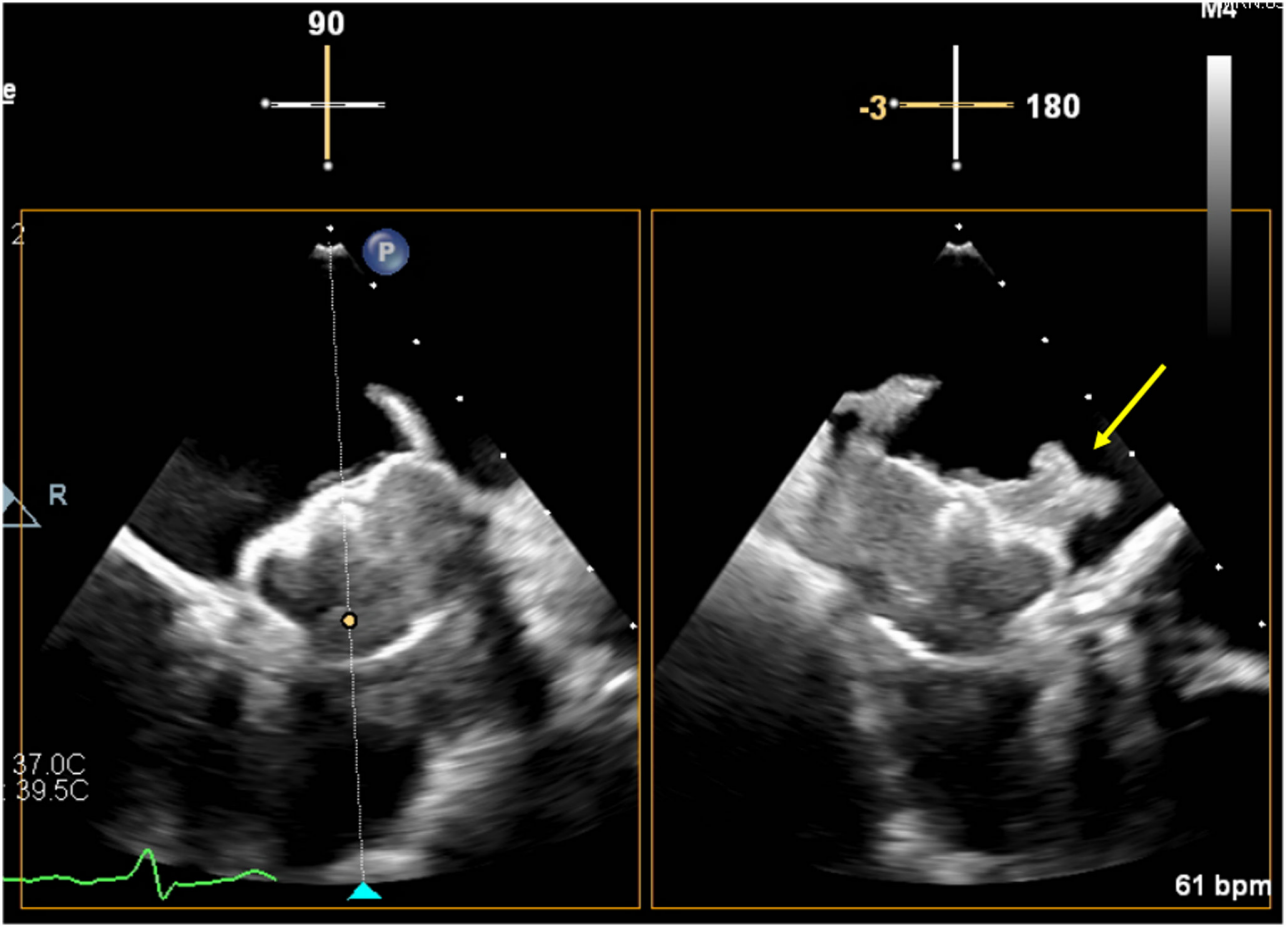
Transesophageal echocardiography image revealing a pedunculated and mobile device‐related thrombosis (yellow arrow) at the time of ischemic stroke diagnosis.

Two months after the resumption of apixaban, a follow‐up TEE showed complete and rapid resolution of DRT. Ten and 16 months after the disappearance of DRT, contrast cardiac computed tomography showed the remaining disappearance of DRT. However, he continued apixaban 5 mg twice daily because of a history of high‐grade DRT, and no bleeding events occurred during the follow‐up period. The antithrombotic regimen for the patient is shown in Figure 3.

**FIGURE 3:**
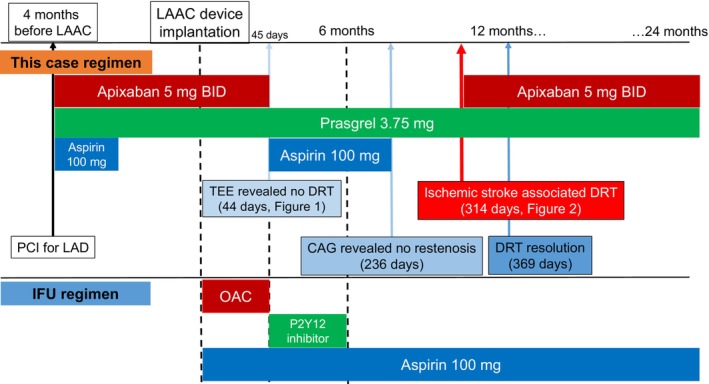
Antithrombotic drug regimen for this patient and instructions for use. CAG, coronary angiography; DRT, device‐related thrombosis; IFU, instructions for use; LAAC, left atrial appendage closure; LAD, left anterior descending artery; PCI, percutaneous coronary intervention.

The pathogenesis of DRT is multifactorial, including patient‐related factors and postprocedural antithrombotic regimens. In this case, he had several risk factors for DRT, including permanent AF, prior ischemic stroke, thromboembolic events despite continuous OACs, a higher CHA_2_DS_2_–VASc score, a large LAA, and the use of a large LAAC device. These characteristics have been identified in the literature as predictive factors for DRT. Among several DRT predictors, the most consistent predictors in terms of medical history were prior ischemic stroke and permanent AF.^2^ There are higher spontaneous echo contrast and lower velocity in such patients, which may cause complete endothelialization delay and DRT. Regarding the post‐LAAC antithrombotic regimen, a previous study have indicated that postprocedural half‐dose direct oral anticoagulants (DOACs) therapy for high‐thrombotic‐risk patients did not cause DRT events and showed both efficacy in preventing thromboembolic events and safety in major bleeding events.^3^ The patient had undergone coronary artery stenting 4 months before LAAC, and although the antithrombotic regimen was complicated, anticoagulation should have been continued in this patient. A previous study showed that LAAC itself did not further reduce the risk of embolism compared with OACs.^4^ Continuation of OACs, including half‐dose DOACs, after LAAC may be a better option for patients at high risk of thromboembolism to prevent further thromboembolic events.^3^ In patients with multiple risk factors for DRT, frequent imaging surveillance, especially in the first year post‐LAAC and after discontinuation of OAC, may be preferred for the early detection and treatment of patients with DRT. In addition, in patients who have suffered an ischemic stroke after LAAC, such as this patient, it is crucial to assess the presence of DRT. If DRT is diagnosed, prompt initiation of appropriate anticoagulation therapy and frequent image surveillance for evaluation are essential. However, the optimal treatment regimen and duration for DRT remain uncertain. Because most DRT were known to resolve within 3 months, imaging surveillance at 1–3 months and 6 months and yearly control after DRT resolution could be appropriate.^2^, ^5^ A recent study reported that thromboembolic events were less frequent in cases where DRT was resolved using appropriate therapy than in those with persistent or recurrent DRT.^5^ It is important to prevent recurrent DRT in patients with a history of resolved DRT. Moreover, long‐term anticoagulation and frequent imaging surveillance are essential to prevent recurrent DRT after DRT resolution.^2^, ^5^ Fortunately, in this case, there were no instances of recurrent DRT or thromboembolic events while maintaining appropriate anticoagulation therapy.

## CONFLICT OF INTEREST STATEMENT

Dr. Kubo is a clinical proctor for Boston Scientific and has received an honorarium from Boston Scientific. All other authors declare no conflict of interest.

## ETHICS STATEMENT

Approval was obtained from the local ethics committee.

## PATIENT CONSENT STATEMENT

Written informed consent was obtained from the patient for this case report.

## Supporting information


Video S1.

Video S2.

Video S3.

Video S4.
